# Enhancing Time-of-Flight Diffraction (TOFD) Inspection through an Innovative Curved-Sole Probe Design

**DOI:** 10.3390/s24196360

**Published:** 2024-09-30

**Authors:** Irati Sanchez Duo, Jose Luis Lanzagorta, Iratxe Aizpurua Maestre, Lander Galdos

**Affiliations:** 1 IDEKO Member of Basque Research and Technology Alliance, 20870 Elgoibar, Gipuzkoa, Spain; jllanzagorta@ideko.es (J.L.L.); iaizpuruamaestre@ideko.es (I.A.M.); 2 Faculty of Engineering, Mondragon Unibertsitatea, 20500 Mondragon, Gipuzkoa, Spain; lgaldos@mondragon.edu

**Keywords:** time of flight diffraction (TOFD), probe validation, weld inspection, ultrasonic (UT)

## Abstract

Time-of-Flight Diffraction (TOFD) is a method of ultrasonic testing (UT) that is widely established as a non-destructive technique (NDT) mainly used for the inspection of welds. In contrast to other established UT techniques, TOFD is capable of identifying discontinuities regardless of their orientation. This paper proposes a redesign of the typical TOFD transducers, featuring an innovative curved sole aimed at enhancing their defect detection capabilities. This design is particularly beneficial for thick-walled samples, as it allows for deeper inspections without compromising the resolution near the surface area. During this research, an evaluation consisting in simulations of the ultrasonic beam distribution and experimental tests on a component with artificially manufactured defects at varying depths has been performed to validate the new design. The results demonstrate a 30 to 50% higher beam distribution area as well as an improvement in the signal-to-noise ratio (SNR) resulting in a 24% enhancement in the capability of defect detection compared to the traditional approach.

## 1. Introduction

Time of Flight Diffraction (TOFD) is a commonly used volumetric ultrasound (UT) based technique that is considered to be a non-destructive testing (NDT) technology [1] that was developed in the 1970s [2]. The technique is based on the diffracted waves that even if they are more subtle than the reflected signals [3] they are capable of detecting discontinuities regardless of their orientation. This contrasts with conventional UT where defects cannot be properly seen if they are not parallel to the probe [4]. It is usually used to identify defects in a wide range of different components, particularly welds for example in steel structures [5], polystyrene pipe joints [6] or even at elevated temperatures [7]. This technique is also effective to detect challenging discontinuities such as those with rough planar defects [8] or flaws with varying orientations and complex geometries [9] among others.

**Figure 1:**
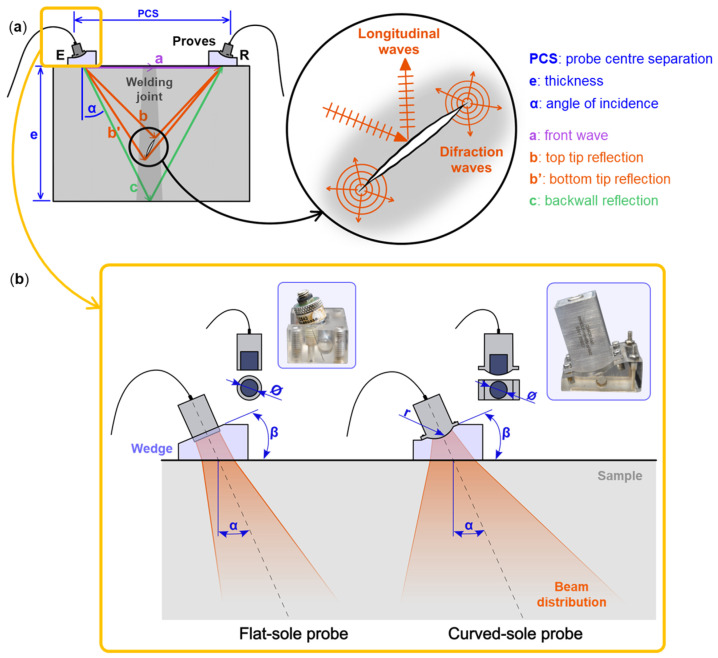
(**a**) Schematic representation of a TOFD system in where the transducers work as emitter and receiver and (**b**) comparison between the usual flat-soled transducer and the novel proposal with a curved-sole as well as the definition of relevant parameters: lens diameter (Ø), angle of incidence (α), wedge angle (β) and the radius of the curved probe (r).

In TOFD, a pair of UT transducers is used to detect discontinuities in the part being analyzed. As shown in Figure 1a, one transducer acts as an emitter (E), emitting short pulsed ultrasonic waves that propagate through the material being evaluated, and the other acts as a receiver (R). The latter registers the signals received: a front wave (i.e., Raleigh wave), which travels along the surface of the sample by the shortest path, and the bottom wave, which bounces off the end of the part. When the emitted wave interacts with an internal discontinuity (e.g., a crack tip, a flaw, the back wall of the part, etc.), part of the wave energy is diffracted and scattered back to the receiver, and the energies of the flaw tips (b and b’) are also recorded [10,11]. By knowing the time of flight of the emitted wave, the discontinuity can be located and sized. Overall, the most important parameter is the distance between the transducers, this is defined as the probe center separation (PCS), and it is set based on the angle of incidence (α) and the thickness (e) of the material to be inspected.

Several attempts have been made to enhance TOFD defect detectability. These include using shear waves to identify defects that are not visible with longitudinal waves [3], performing TOFD in immersion to improve inspection of lower thicknesses [12,13], exploring methods to detect fatigue damage with TOFD techniques [14], and implementing narrow pulse transducers for thick wall analysis [15]. Remarkably, a custom array transducer to improve the lateral resolution of TOFD images has also been developed [16]. This approach allows for better detection and characterization of defects by enhancing the clarity of the captured images but just focuses on the improvement of shallow defects. The reduction of the dead zone by using mode-converted waves and advanced signal processing has also been achieved, improving the detection of shallow subsurface defects [17] on thin samples. Notably, a TOFD technique using multiple receiving transducers and source-positioning algorithms for 3D defect localization has also been developed [18]. Experimental results showed high promise, with potential for increased accuracy using more transducer pairs. However, this approach complicates the scanning phase and doesn’t guarantee broader defect detection compared to traditional methods. Interestingly, simulation models have also proven to be powerful tools for optimizing TOFD inspection techniques. By leveraging an understanding of the underlaying physics, simulations have enabled reliable assessments of structural integrity [19] and accurate predictions of diffraction echoes from complex 3D flaws, thereby aiding in the characterization of disoriented cracks [20].

In addition, efforts have also been made to improve the post-processing of TOFD tests in an attempt to automate this step [21]. It is noteworthy that the work that has been done in where a neural networks based machine learning post-processing SW has been developed to analyze the signals [22]. In the same direction, in [23] the inspection speed and accuracy were improved by integrating automated signal and image processing techniques that are capable of identifying and sizing defects measured with usual TOFD transducers. Moreover, Full Matrix Capture (FMC) and Total Focusing Method (TFM) techniques have also been applied to TOFD data to help correct for diffraction effects, resulting in more accurate defect sizing and positioning [24].

However, even with the recent achievement, the usual TOFD technique has its shortcomings, particularly when inspecting thick samples: a simple, fast and reliable way of inspecting defects throughout the entire thickness of a wide sample without using multiple scanning methods, is still lacking. According to the ISO 10863:2020 standard “Non-destructive testing of welds—Ultrasonic testing—Use of time-of-flight diffraction technique (TOFD)” [25] it is required to utilize at least two different setups when inspecting a component with a thickness exceeding 50 mm to ensure an accurate assessment across the full width. Consequently, this leads to an increase in both time and resources required for the examination. With the aim of analyzing thick walls in a single pass by increasing the detection capabilities of TOFD transducers an innovative design was proposed and patented by the authors [26]. This novel design is based on a complex curved sole instead of the traditional flat sole approach. This probe idea widens the focusing area by improving the conventional design, enabling the inspection of thick samples (i.e., over 100 mm) with just one set up and pass. This paper presents a comprehensive comparative analysis of conventional flat-sole probes versus the effect of applying a curvature to the TOFD transducers sole, see Figure 1b. The primary objective is to understand the underlying physics of the curved sole design and to demonstrate its advantages over traditional methods in detecting discontinuities, particularly in deep inspection zones, thereby enhancing the effectiveness and efficiency of the process. The study examines the impact of probe shape, lens diameter, wedge angle, and PCS to determine the optimal parameter range for this new design.

## 2. Materials and Methods

This section outlines the methodology employed in the investigation, which aimed to compare flat and curved-sole probes. Firstly, the beam distribution of both transducer types has been analyzed through simulation models using the “Extende CIVA 2023” software. Secondly, experimental tests have been conducted to prove the performance of the transducers in a pattern block with known defects. For the interpretation of the results the signal to noise ratio (SNR) was calculated for each identified defect. This dual methodology, blending simulation and experimentation, ensures a thorough comparison of the flat and curved-sole probes and provides the perfect way to understand the behavior of the ultrasonic beam in this new approach.

### 2.1. Definition of the Flat and Curved-Sole Probes

The transducers used in the study, for both in the simulations and the experiments, had the following characteristics:

- Flat probes: A pair with a Ø6.36 mm lens diameter, operating at 5 MHz and 200 V, with a bandwidth of 112% at −6 dB.
- Curved probes: Two pairs with a sole curvature radius of 40 mm, operating at 5 MHz and 200 V, with a bandwidth of 95% at −6 dB. The effect of the lens diameter was analyzed, with one pair featuring an Ø8 mm lens and the other an Ø12 mm lens.

This choice was made because the 6.36 mm diameter of the flat probe has been shown to provide an optimum balance between acoustic pressure and coverage area, which is critical to maintaining resolution. For the curved probes, larger diameters (i.e., 8 mm and 12 mm) were selected to counteract the natural spreading of the ultrasound beam caused by the curvature. This spreading reduces sound pressure, making it harder to detect deep or small defects in thick walled samples. By using larger diameters, the area of acoustic pressure is increased, ensuring that the curved probes maintain effective detection capabilities at greater depths, despite the beam spreading. On the other hand, a larger lens diameter on a flat probe, such as 8 mm or 12 mm, would reduce the divergence angle of the ultrasound beam, leading to a narrower beam that decreases the coverage area and potentially worsens the resolution, particularly when trying to detect small or deep defects. Therefore, increasing the lens size in flat probes would be counterproductive for the goals of this study. These diameter choices ensure that each probe type is optimized for its specific application, providing the best possible performance and a fair comparison.

Moreover, in TOFD methodology, achieving precise probe focusing is crucial, which is why angled wedges are used. These wedges are shaped to match the exact contour of the probe, ensuring a seamless fit between the transducer and the part. The flat probes are set at 60° and 70°, whereas the curved probes conform to the slanted wedges positioned at 40° and 60°. The flat transducers and their wedges were purchased from the OLYMPUS catalogue, while the curved set were designed by IDEKO [26].

### 2.2. Simulation Model: Study of the Beam Distribution

To understand the beam distribution of each probe, simulations were performed using the “beam calculation” mode in CIVA 2023. The analysis was conducted considering the parameters described in Section 2.1 and for various PCS, ranging from 100 mm to 400 mm in 50 mm increments. The summary of all the used configurations is shown in Table 1.

The same carbon steel block pattern used in the experiments was modelled, considering a sound propagation velocity of 5900 m/s for longitudinal waves and 2680 m/s for the wedges made of perplex. Both fitting interfaces (i.e., probe/wedge and wedge/part) were assumed to be perfect. To ensure smooth sound propagation between different interfaces, oil was used as a coupling material between the probe and the wedge, while distilled water was used between the wedge and the part.

**Figure 2:**
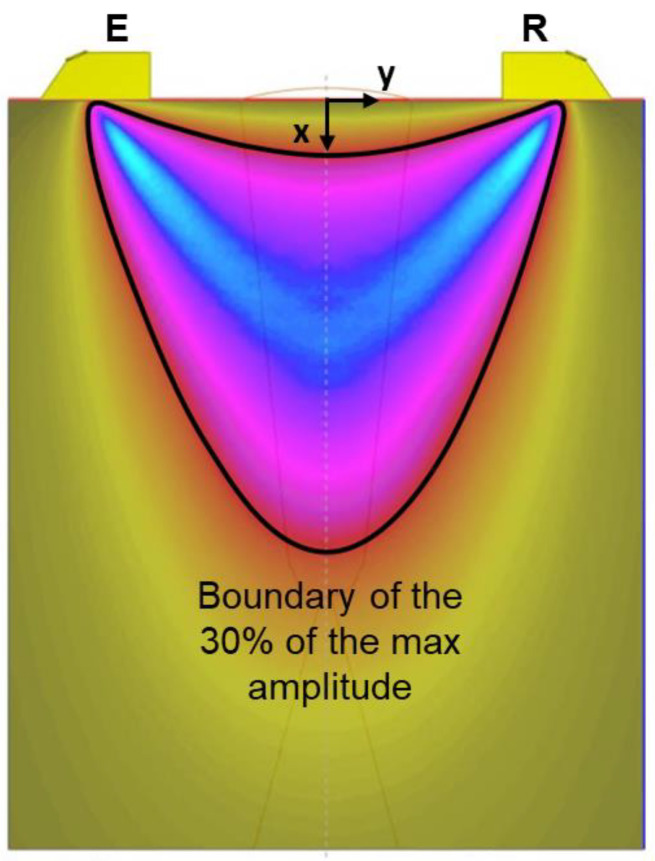
Example of the methodology of the beam distribution analysis of longitudinal waves performed in “Extende CIVA 2023” (Curved-sole probes, Ø12 mm, 60° and 160 mm PCS).

To study the beam distribution of the longitudinal waves, each configuration was methodically run in batch mode in CIVA, varying one parameter per iteration. The acquired data was later post-processed using Python 3.10.14. For each simulation, the amplitude of the acoustic beam at the cross-sectional plane of the scan was determined. A contour boundary was then established at 30% of the max amplitude, see Figure 2, outlining the region where longitudinal waves are concentrated (i.e., the useful area of the beam in where the discontinuities are detected). This method provided a standardized baseline for evaluating the performance of each probe configuration. If any PCS is not visible in the graph, it means that the maximum sound pressure under those conditions is lower than the defined threshold.

### 2.3. Experimental Set Up: Study of the Flaw Detectability

**Figure 3:**
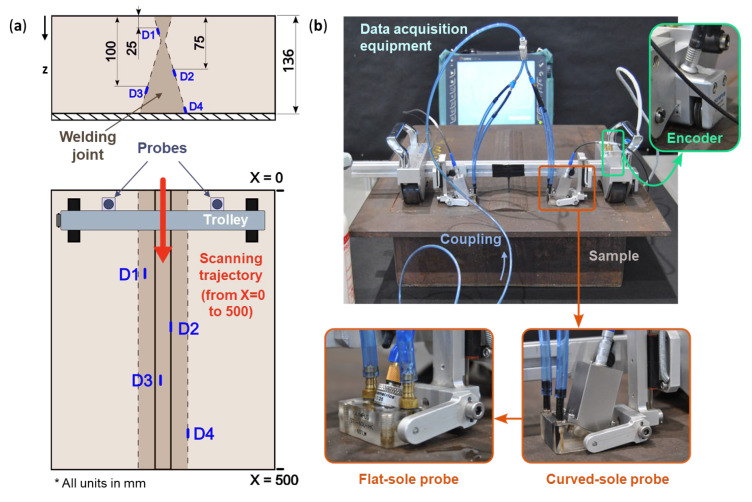
(**a**) Schematic representation of the analyzed part (carbon steel, 136 mm deep, 4 pre manufactured defects (D1–D4) each one 5 mm in depth, in defined locations) as well as the demonstration of the experimental procedure and (**b**) experimental set up depicting the sample, manual trolley with the encoder, coupling and data acquisition system for flat and curved-sole probes.

The component depicted in Figure 3a was employed for the experiments. This 136 mm thick welded junction of two carbon steel plates has four artificially manufactured defects at different orientations that are 2 mm wide, 5 mm tall and 20 mm long that resemble typical welding anomalies.

The testing was conducted with an ergonomic trolley that allows for the use of both flat and curved-sole probes. This trolley ensures a steady motion of the probes, maintaining a constant PCS while being equidistant from the center of the part (i.e., the weld line). Additionally, the trolley provides an uninterrupted supply of distilled water as coupling fluid between the wedges. The trolley was operated manually at a continuous speed of less than 60 mm/min. For data acquisition purposes, an OLYMPUS OMNISCAN MX2 was employed, with an encoder mechanically connected to the trolley to accurately record its displacement. For data processing, the OmniPC 5.9 software was used. This configuration is illustrated in Figure 3a,b.

For the tests the trolley was scanned along the entire length of the sample, parallel to the welding line. These tests were conducted in accordance with the ISO 10863:2020 standard for TOFD, which specifies that the lateral wave echo should be at 80% amplitude to be considered optimal. Moreover, three repetitions of each configuration were documented, and the outcomes shown represent the average of these repetitions. The parameters employed in the analysis of the beam distribution are summarized in Table 2. In this case the PCS parameters differ from the simulation ones to have a higher resolution in the area of interest for the curved sole proves (between 100–200 mm), and points with non-relevant results have been removed for clarity.

## 3. Results

### 3.1. Model Results: Study of the Beam Distribution

**Figure 4:**
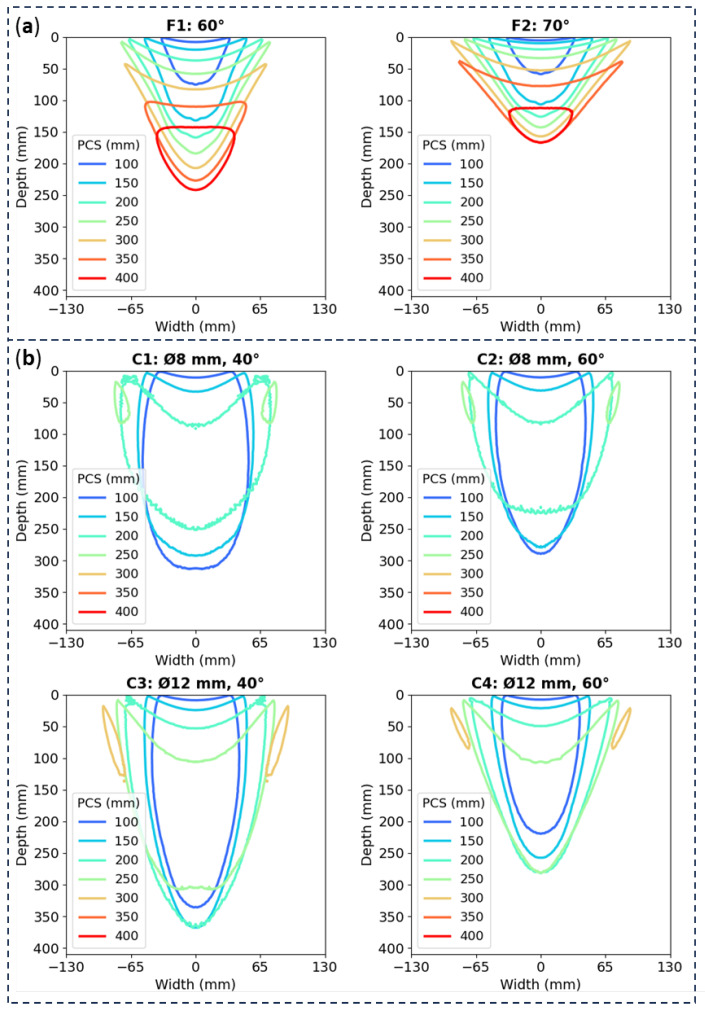
Plot profile comparing the effect of the different PCS values on each set up: (**a**) for flat-soled probes and (**b**) for curved-sole ones.

This section presents the results of a comprehensive investigation into the behavior of longitudinal waves. The examinations carried out in a carbon steel sample were designed to explore all the described configurations with the aim of comparing the acoustic pressures (i.e., effective area of discontinuity detection) regarding the effect of the PCS in both flat and curved-soled probes. The obtained results are presented in Figure 4a,b for the flat and curved probes, respectively. They are organized by PCS and juxtaposed by configuration for a comparative analysis.

The main parameters to compare include not only the sole geometry but also the wedge inclination, the effect of the lens diameter, and especially the Probe Centre Separation (PCS). In our case the wedge inclination and the lens diameter are fix parameters hence the importance of selecting probes that are tailored to specific inspection requirements. On the other hand, the PCS is easily adjustable during the test to suit the optimal requirements and thus it presents the need to be calibrated once the other parameters are set up. Independently of the sole geometry, in the performed simulations it can be observed that the effect of the wedge angle, the lens diameter size and the PCS are as follows:

- The wedge angle is inversely proportional to the angle of incidence of the ultrasound beam. As the wedge inclination increases, the depth of the focusing zone decreases.
- Although it is not the most crucial parameter, the lens diameter size also affects the versatility of the PCS range. A smaller lens diameter, such as Ø8 mm, limits effective focusing to PCS values over 200 mm. Conversely, increasing the lens diameter broadens the range of useful PCS, with a Ø12 mm lens allowing effective focusing up to 250 mm.
- The PCS is the most influencing parameter and makes a great influence in the size of the focusing zone (i.e., the area of high acoustic pressure where discontinuities are best detected). A higher PCS results in a wider and deeper area, although the edge of the area becomes so weak, with only the center remaining above 30% of the max amplitude. Beyond this point, the area begins to narrow again while continuing to deepen.

When comparing flat and curved-sole probes, it is evident that the largest effective PCS ranges for both angles and flat probes are around 300 mm, while for curved probes, the range is approximately 150 mm. Furthermore, for optimal PCS setting, the focusing area of the curved-sole probes is 30 to 50% larger than that of the flat probes, considering the best PCS ranges in each scenario. This significant enhancement in the preferred detection region is a crucial advantage that guarantees a more comprehensive defect detection capability in practical applications. Curved-sole probes can cover a larger and wider scanning area in a single pass, allowing them to reach deeper effective areas while still detecting shallower discontinuities. This versatility is crucial for identifying flaws in thick components. Given these factors, the simulations suggest that the curved-sole probes are a significantly more promising option for enhancing ultrasonic flaw detection compared to standard flat probes.

### 3.2. Experimental Validation: Study of the Flaw Detectability

**Figure 5:**
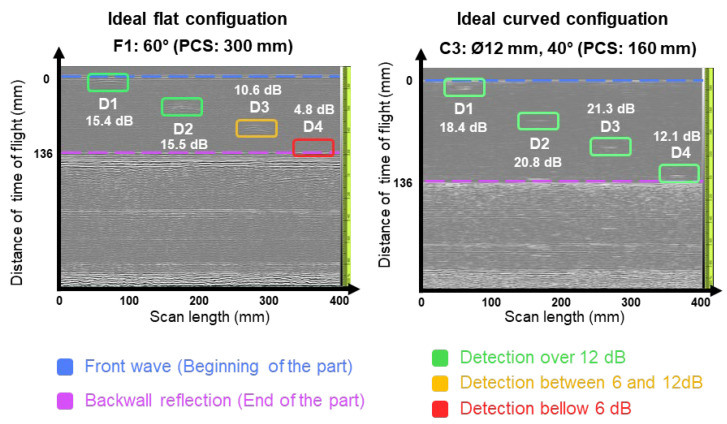
D-scans from the optimal configurations. F1 and 300 PCS for flat sole probe and C3 and 160 mm PCS for curved sole probe.

In this section, experimental tests are presented to verify the promising assumptions made in the simulations of the behavior of the acoustic beam. Figure 5 illustrates the D-scans of the ideal configuration of flat and curved probes from the experimental tests, with the x-axis representing the scan length and the y-axis indicating the distance of the time of flight. Interestingly, while the 160 mm PCS configuration proves to be non-ideal for flat probes, it is effective for curved ones, while the 300 mm PCS configuration shows the opposite trend. This contrast underscores the importance of selecting an appropriate PCS for optimizing probe performance. An analysis of effective PCS cases between flat and curved probes demonstrates the advantages of the curved design. Unlike the flat probe, which only identifies three out of four defects, the curved probes are also capable of identifying the deepest ones.

To quantitatively evaluate the TOFD diffraction results, a signal-to-noise (SNR) calculation was performed for each defect in every scan following the Equation (1). The average signal value as well as the average noise value are obtained by measuring a representative square from the defect and next to the defect respectively.

(1) $${SNR}_{dB}=20\;\log_{10}\left(\frac{Average\;Signal\;Power}{Average\;Noise\;Power}\right)$$

**Figure 6:**
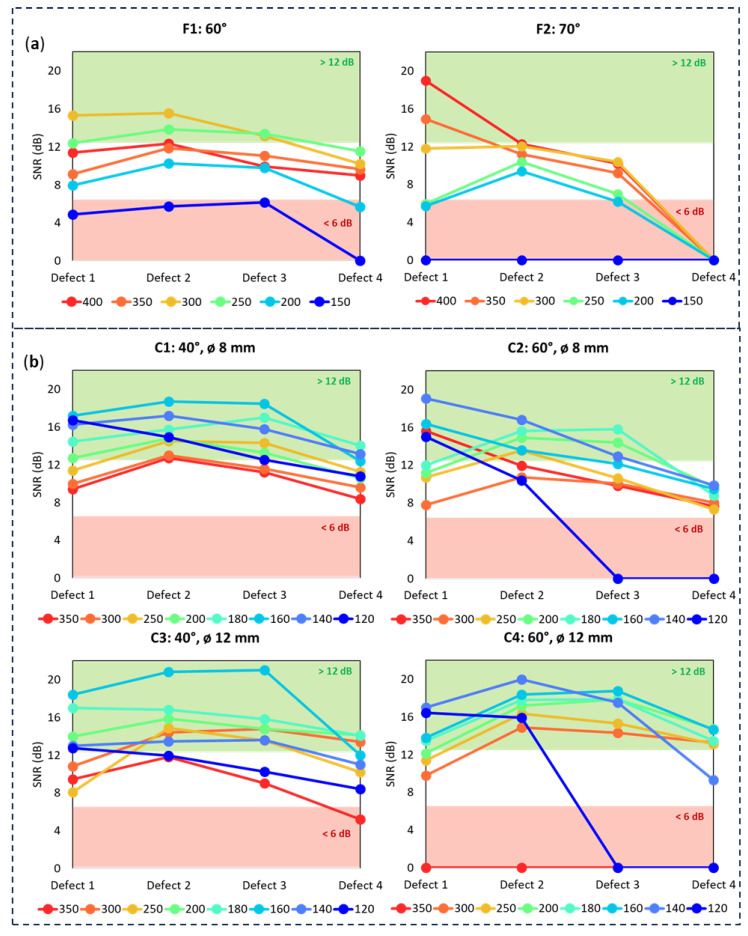
Plot profile comparing the SNR mean values considering the effect of the different PCS values and defect depths on each set up: (**a**) for flat probes and (**b**) for curved ones.

The SNR values were then compared across different configuration and PCS settings. The resulting data has been systematically organized by configurations: Figure 6a for flat-sole probes and Figure 6b for curves ones. Within each graph distinct plots are represented for individual PCSs and defects. The results assigned to the defects represent the average of the three consecutive scan repetitions with a mean variation of 0.8 dB, hence providing a robust basis for comparative analysis across configurations.

Here it can be seen that, in the best case scenarios, the SNR curves exhibit an improvement of 24% for curved sole probes compared to flat-sole probes. Specifically, for flat probes, the optimal PCS ranges from 300 to 350 mm, yielding an average SNR of 15.5 dB. In contrast, curved-sole probes, with an optimal PCS range of 160 to 180 mm, can achieve a remarkable 21.3 dB. This dB difference is significant for defect detection. The higher mean SNR, which can exceed +6 dB in some cases, indicates that curved-sole probes capture a more robust acoustic signal, enhancing their effectiveness in identifying defects.

### 3.3. Discussion

**Figure 7:**
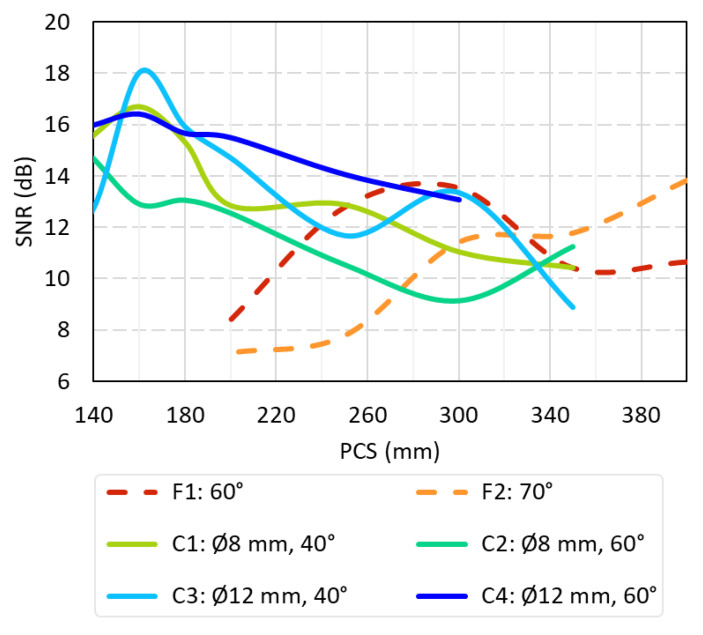
Summary of the SNR results for each configuration depicting an average per defect and repetition and divided by configuration and PCS: dashed lines for flat-sole probes and continuous for curved-sole ones.

Overall, the summary of the SNR results for each configuration is shown in Figure 7, where the trends by PCS are visually displayed and the results obtained through the research are clearly visible. Here, dotted lines denote the flat-sole probes, whereas solid lines indicate the curved-sole ones. Notably, the optimal analysis parameters leading to the highest SNR results are observed at approximately 300 mm PCS for the 60° angled flat-sole probes and over 350 mm PCS for the 70° counterparts. The curved-sole configurations display optimal signal-to-noise ratio (SNR) values between 160–180 mm PCS. Moreover, it is evident that well-calibrated curved-sole probes consistently produce higher SNR measurements than their counterparts. This observation aligns with the outcomes of the beam distribution analysis in the simulations, reinforcing the correlation between probe sole geometry and configuration, calibration precision, and signal-to-noise ratio performance.

This comparison clearly demonstrates that several curved sole prove configurations (specifically, the combinations of 40° wedge angle with both diameters and 60° angle with the Ø12 mm) has an improved outcome in comparison to the flat probes. This result is aligned with the simulation results shown in Section 3.1/Figure 4: which showed that the beam distribution area is 30 to 50% wider than that of flat-sole probes across all tested combinations. This highlights the enhanced versatility of the new design.

Increasing the acoustic signal by approximately 6 dB provides a range of significant benefits in improving the defect detection [27]. The improved sensitivity resulting from higher SNR enables curved-sole probes to detect smaller defects with greater resolution and sensitivity, surpassing the capabilities of their flat-sole probes and providing a substantial safety margin. Particularly in challenging conditions characterized by higher background noise, greater defect depths, thicker samples or materials with attenuating properties [28]. Moreover, the reduction in false negatives, a notable outcome of the higher SNR, minimizes uncertainties and human error, boosting the reliability of defect identification, mitigating the risk of inaccurate results and improving operators’ working conditions [22,23]. This enhanced acoustic signal not only ensures consistency and reliability in defect detection but also contributes to the robustness of the entire detection system.

Finally, curved-sole probes provide an optimal PCS that is shorter than flat probes (i.e., curved PCS ≈ 160–180 mm while flat PCS ≈ 300–350 mm). This new probe design allows to create devices accessible to more restrictive components. This has extensive advantages such as the reduction of preparation area, increased accessibility in confined or challenging spaces, and potential for a lighter and shorter carriage to facilitate easier handling and transportation. Curved-sole probes offer increased flexibility, allowing for a wider inspection range. Moreover, the fact of having lighter and shorter inspection tools combined with the capability of inspecting the full width of the part in a single pass contrary to the multiple passes required for parts over 50 mm makes the automatization of the process more manageable.

## 4. Conclusions

Overall, this study proposes a new transducer design featuring a curved sole for Time-of-Flight Diffraction (TOFD) analysis, aimed at enhancing defect detectability. The research compares the newly designed curved-sole transducers to conventional flat-sole ones, employing simulations to observe the beam distribution as well as empirical testing.

Simulations of beam distribution with the new curved sole demonstrate a 30 to 50% larger focusing area, where high acoustic pressure enhances the detection of discontinuities, compared to the flat-soled ones. Experimental tests have supported simulation results by confirming that curved-sole probes provide a 24% increase in SNR (i.e., over +6 dB) when compared under equivalent conditions. This demonstrates that curved-sole probes are capable of capturing a more robust acoustic signal, allowing for the detection of smaller and deeper defects at a wider depth, granting a greater resolution and sensitivity.

Moreover, this test has also demonstrated that PCS is the most influential parameter, compared to the refraction angle and lens diameter, with curved-sole probes performing optimally at shorter distances. Specifically, curved-sole probes are most effective with a PCS of 160 to 180 mm, whereas flat-sole probes perform best with a PCS of 300 to 350 mm. This allows for the development of smaller and lighter designs that are easier to handle and more suitable for accessing confined spaces.

It is clear that these new transducers offer a larger and more versatile scanning area, outperforming flat probes in terms of defect detection and SNR. They boost TOFD capabilities, thus improving non-destructive defect detection testing especially in thick-walled materials and provide a more efficient solution for ultrasonic flaw detection in practical scenarios.

## Figures and Tables

**Table 1:** Summary of the simulated configurations.

Set Up Nº	Type of Probe Sole	Lens Ø (mm)	Wedge Angle (°)	PCS (mm)
F1	Flat	6.36	60	100, 150, 200, 250, 300, 350, 400
F2	Flat	6.36	70	
C1	Curved	8	40	
C2	Curved	8	60	
C3	Curved	12	40	
C4	Curved	12	60	

**Table 2:** Summary of the experimental test configurations.

Set Up Nº	Type of Probe Sole	Lens Ø (mm)	Wedge Angle (°)	PCS (mm)
F1	Flat	6.36	60	150, 200, 250, 300, 350, 400
F2	Flat	6.36	70	
C1	Curved	8	40	120, 130, 140, 160, 180 200, 250, 300, 350
C2	Curved	8	60	
C3	Curved	12	40	
C4	Curved	12	60	

## Data Availability

Data are contained within the article.
